# Professional medical education approaches: mobilizing evidence for clinicians

**DOI:** 10.3389/fmed.2023.1071545

**Published:** 2023-07-28

**Authors:** Xiafei Lyu, Sheyu Li

**Affiliations:** ^1^Department of Radiology, West China Hospital, Sichuan University, Chengdu, China; ^2^Department of Endocrinology and Metabolism, West China Hospital, Sichuan University, Chengdu, China; ^3^Division of Guideline and Rapid Recommendation, Cochrane China Center, MAGIC China Center, Chinese Evidence-Based Medicine Center, West China Hospital, Sichuan University, Chengdu, China

**Keywords:** professional education, continuing medical education, evidence mobilization, dissemination strategies, clinicians

## Abstract

Rapidly proliferating high-quality evidence supports daily decision-making in clinical practice. Continuing professional medical education links this evidence to practicing clinicians who are strongly motivated to improve the quality of their care by using the latest information. Approaches to professional education vary, and their effects depend on specific scenarios. This narrative review summarizes the main approaches for professional medical education that facilitate the mobilization of evidence for clinicians. It includes traditional learning (passive and active dissemination of educational materials, lectures, and mass media dissemination), constructivist learning (engaging in local consensus processes and education outreach visits, interfacing with local opinion leaders, conducting patient-mediated interventions, employing audit and feedback processes, and utilizing clinical decision-supporting systems), and blended learning approaches (the integration of in-person or online passive learning with active and creative learning by the learners). An optimized selection from these approaches is challenging but critical to clinicians and healthcare systems.

## 1. Introduction

A vast and increasing body of literature spurs rapid growth in medical science, but clinicians grapple to remain abreast of the vast quantities of rapidly proliferating publications. The resulting information overload can overwhelm and confuse healthcare providers, especially those who are unable to discern credible research from low-quality output (1, 2). Publishing evidence in journals or issuing guidelines does not guarantee changes in practice (3, 4). As such, the process of translating the ever-growing body of clinical evidence into practice is suboptimal (5–7).

Clinical practice guidelines are major bridges that link evidence and practice. However, only an average of 67% of medical decisions is made based on the guidance documents (8). Both clinicians and clinical epidemiologists complain about the translation of data from evidence into real-world practice (9). While barriers to the absorption and implementation of new knowledge vary, a lack of sufficient time among clinicians is the most prevalent and dominant obstacle (10), followed by a lack of awareness regarding evidence collection and appraisal, limitations in library sources (10), and inertia (11, 12). In such circumstances, clinicians seek trustworthy and easy-to-follow sources of information to keep their practice up-to-date (1).

Continuing professional medical education grounded in evidence-based materials provides a platform for skill and knowledge promotion among practicing clinicians (13). By learning about high-quality evidence and trustworthy clinical practice guidelines or interpretations, clinicians update care regimens that improve patient outcomes. In contrast, flawed or misleading information impairs decision-making.

Numerous strategies have been developed to improve the effectiveness of teaching. Several taxonomies have also been generated to combat the lack of conceptual clarity regarding different strategies covered in the published literature, including the Cochrane Effective Practice and Organization of Care (EPOC) taxonomy and the Expert Recommendations for Implementing Change (ERIC) taxonomy. While the EPOC taxonomy provides a practical way to identify implementation strategies targeted at healthcare professionals (14), the ERIC taxonomy is a more comprehensive compilation that summarizes 73 discrete dissemination and implementation strategies and provides a list to healthcare implementation scientists (15).

### 1.1. Instructional models

**Figure 1:**
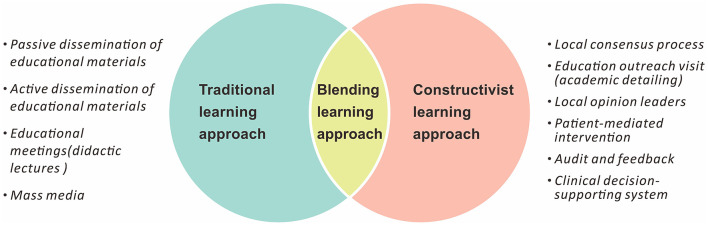
Summary of the approaches for professional education.

Table 1 and Figure 1 illustrate the common instructional models including traditional, constructivist, and blended learning models. Instructors who focus solely on the simple passive transmission of information from educators to learners are considered to be working within a “traditional learning” model, for example, a conventional classroom (16). Alternatively, in constructivist models, learning is considered as an active process, and knowledge is co-created between individuals. In constructivist learning, the knowledge is constructed in a way that makes sense of learners' experiences and modifies the learners' existing beliefs in order to reduce the amount of cognitive dissonance (17). Blended learning is one of the modern learning techniques that integrate/in-person or online passive learning (traditional learning approaches) with active and creative learning by the learners (constructivist learning) (18).

**Table 1:** Summary of the main approaches for professional education.

**Category 1: traditional learning**			
**Approach**	**Characteristics**	**Advantages**	**Disadvantages**
Passive dissemination of educational materials	Presenting educational materials in easy-to-read format, such as clinical practice guidelines, color print, newsletters, et al.	High readability and a clear structure Alignment with the reading habits of the audience.	Readers may ignore important details or supportive text elaborating on recommendations.
Active dissemination of educational materials	Distribution of materials through personal delivery or posts to the Internet.	The most commonly used approach.	The efficiency of this strategy depends on its source, channel, and format.
Educational meetings (didactic lectures)	Courses, seminars, workshops, etc.	Widely used for continuing medical education It summarizes large amounts of well-established information It can be tailored-made based on a given situation.	Possibility of high costs. Industry-funded events raise conflicts of interest concerns.
Mass media	Radio, newspapers, leaflets, posters, booklets, alone or in conjunction with other interventions. Targeted at the population level.	Dissemination efficiency is high. It can reach large number of people.	Significant potential conflicts of interest that request auditing and surveillance. The cost of mass media is very high and is unlikely to be covered by public funds.
**Category 2: constructivist learning**			
Local consensus processes	Participating clinicians discuss and endorse both a problem of importance and evidence-based solution.	Benefit for reforming local practice and health equity improvement.	It often requires involvement from local medical societies and clinical epidemiologists.
Educational outreach visits	Using clear educational and behavioral objectives, trained clinical educators deliver face-to-face encounters within practice settings.	Educational content is tailored-made to clinicians. Information can reach remote and rural areas.	Visits can be expensive. Funding from private sources raises conflicts of interest risks.
Local opinion leaders	Opinion leaders (selected formally or informally) deliver and manage information for clinicians.	These leaders are well-regarded and influential among clinicians. They offer experience relevant to practice.	Identification of these leaders is critical. Some may be biased and under-qualified.
Patient-mediated intervention	Any intervention aimed at changing the performance of clinicians through interactions with real or standard patients or information provided by or to patients.	It enhances patients' knowledge about their condition and support their role in decision-making, which in turn can encourage more active self-management.	This methodology is new in China and other developing countries. Its adoption may need time for training standard patients to effectively engage clinicians and the health system.
Audit and feedback	Any summary of the clinical performance of healthcare over a specified period of time aimed at providing information to health professionals to allow them to assess and adjust their performance.	It works more efficiently among those with lower baseline performance and when feedback is delivered more intensively.	Audit and feedback are not suitable for targeted behavior with a high degree of complexity.
Clinical decision-supporting system (CDSS)	A CDSS is embedded within electronic health records. It sends reminders on those episodes at the point of appropriate time.	The CDSS sends reminders on those episodes at the point of care at the appropriate time.	The development of the CDSS is difficult for it calls for close cooperation between medical and computer science.
**Category 3: blended learning**			
Blended learning	Online learning modules plus live face-to-face learning. Online learning provides background information and sets the stage for the interactive case materials that follow. An expert-supervised in-person workshop or training task enhances the practical skills of learners.	Clinicians can learn on their own time without the inconvenience of travel. Room is allowed for creative and cooperative exercise.	High cost is a critical challenge. The quality of the virtual lectures is also a large concern.

### 1.2. Funding bodies and conflicts of interest

Conflicts of interest are inevitable in real-world educational and clinical scenarios (19). They impact the quality of an educational program but are easy to ignore in practice. Industries, including but not restricted to those in the pharmaceutical field, often offer continuous medical education that may influence the decision-making of clinicians. However, this involvement raises serious concerns (19, 20), as inherent conflicts of interest could introduce bias into professional medical education (21).

Accreditation systems are devised to guarantee the credibility of educators and determine that delivered materials are without bias (21). In Europe and North America, accreditation councils for continuing medical education require sponsors to be transparent regarding their roles in educational activities (20, 22). The amount of industry funding for specific professional education activities must also be disclosed (19). In cases of bias, the council or other organizations could suppress materials to prevent poor medical behavior in practice (19). Given their non-profit nature, many medical professional societies are the most appropriate bodies to hold or fund professional educational programs.

## 2. Method

We searched Pubmed for studies about continuing medical education from January 2012 to October 2022. The search terms for titles, abstracts, or MeSH terms included “guidelines” or “recommendations” and “disseminat^*^” or “information dissemination” and “healthcare” or “clinicians,” “continuing medical education,” or their synonyms. Searches were not restricted by language or publication type. The authors added gray literature with their expertise. We also browsed the reference lists of narrative reviews of the interests that were identified in the literature search.

## 3. Traditional learning approaches

### 3.1. Passive dissemination of educational materials

The presentation of educational materials in easy-to-read formats is critical to their passive dissemination among clinicians. Some examples of effective formats include clinical practice guidelines presented in modular knowledge chunks, flowcharts, and abstracted infographics. Although color printing is not new, it remains effective in making hard copies more attractive to audiences. Shorter-form newsletters, bulletins, monographs, and reprints may also be more user-friendly for readers. However, they can also be biased, particularly if they come from organizations with conflicts of interest (10, 23–26).

Clinical practice guidelines are popular materials from the perspective of most clinicians. Posting such information through print and online media attracts clinicians' attention and enhances its spread (27). The presentation of such guidelines evolves with reading habits (10, 28).

The modular knowledge chunk format allows the guideline recommendations to be packaged into distinct chunks of information for individual disease related topics. They often consist of a summary table, a brief synopsis, and separate supportive text elaborating on each recommendation in detail (10, 29).

Other methods for passive communication include flowcharts, which are also commonly adopted for new guidelines and are often considered essential to reporting (30, 31). Translation into multiple languages promotes the dissemination of evidence among different countries (30). Electronic versions of publications make them easier to access. By placing an abstracted infographic at the top of the front page (e.g., BMJ Rapid Recommendations), fast access to information and supporting evidence is facilitated (25, 32, 33).

### 3.2. Active dissemination of educational materials

The active dissemination or mobilization of educational information is one of the most common approaches for clinical practice guidelines, monographs, publications in peer-reviewed journals, audiovisual materials, electronic publications, and other materials (34). This strategy involves either person-to-person email or internet posts (14, 35) and mildly improves the performance of healthcare materials (24, 34). The efficiency of an active dissemination strategy depends on the source, channel, and format (35, 36). Sources of educational materials vary, but published research and guidelines are usually the top choices. A systematic review reported that active dissemination of educational material resulted in a 2% improvement in professional practices when compared to no intervention (24, 37).

Some academic institutions and hospitals may also contribute to the material's dissemination, with benefits to career development, rankings, and funding sources. The academic reputation of the disseminators guarantees the quality of the materials. While pharmaceutical industries are also passionate about sharing their funded studies, scholars and members of the public remain dubious of these sources due to potential conflicts of interest (35). A journal publication itself is also a platform for dissemination. Nevertheless, only 62% of clinicians screen academic journals more than five times per week (38). Point-to-point delivery and social media (such as Twitter and WeChat) are widely used and considered helpful (35, 39). The frequency of delivery could be once, twice, three times, or more per week, and it determines the strength and cost of the dissemination process (35, 40). Regional, national, and international conferences also facilitate the distribution of educational materials (10).

### 3.3. Educational meetings (didactic lectures)

Educational meetings are common for disseminating well-established, clinically relevant information to healthcare professionals (41). These meetings are typically courses, seminars, and, in some cases, workshops. The nature of educational meetings varies in aim, targeted practice, length, frequency, content, capacity, and type of interaction (41). Educational meetings are effective, with a systematic review reporting that their use can increase clinicians' adherence to desired behaviors by ~6% when compared to no intervention (42). The performance of educational meetings is scenario-specific and requires a tailored plan based on a given situation. Feedback collected from the audience improves the future performance of meetings at little to no extra expense.

Educational meetings can be very costly, especially when they involve big conference halls with state-of-the-art equipment or famous speakers. High prices prevent regular high-quality meetings from being held by medical bodies without adequate funding. Although industry-funded educational meetings are common, their credibility is a major concern due to potential conflicts of interest.

### 3.4. Mass media

Television, broadcast, and newspapers may be helpful to professional medical education (14). Mass media, with its power of rapid and global transmission, can open up unprecedented opportunities for evidence dissemination. Nevertheless, the cost of mass media is very high and is unlikely to be covered by public funds, but it is preferred by the industry. Given the significant risk of conflicts of interest, the content of these forms of media needs critical auditing and surveillance.

## 4. Constructivist learning approaches

### 4.1. Local consensus process

In a local consensus process, a discussion takes place among participating clinicians who reach an agreement that a chosen clinical problem is important and the evidential approach to managing the problem is appropriate (14). Subsequent meetings can facilitate a community-based consensus on treating a disease or adapting external guidelines (most of them promulgated on the national or international level) to fit the local setting, thus improving compliance. Compared to a control group, a small-group consensus process increased the participants' adherence to influenza vaccination guidelines by ~34% (43). In addition to the agreement formed by participating clinicians, the process could concurrently reform local practice and improve health equity in the community (14). It should be noted that for most communities, such processes necessitate an organizational effort from local medical societies with strong influence from clinicians, as well as technical assistance from clinical epidemiologists (43).

### 4.2. Educational outreach visit

During educational outreach visits (also known as academic detailing), a group of trained clinical educators delivers work with clinicians in their practice settings (44). This promising approach is to modify the practice of clinicians, in particular prescribing. A systematic review demonstrated that, compared with no intervention, outreach visits could increase clinicians' compliance with desired behaviors by ~20–50% (45).

The success of an educational outreach visit depends on the level of training of the detailers. When experienced educators are not available, pre-program training may be necessary to help them build cultural and knowledge backgrounds and communication skills (46). The National Resource Center for Academic Detailing (NaRCAD; www.NaRCAD.org) offers examples for such preparation and summarizes key components of detailing including introduction, needs assessment (or motivation interview), key messages, objection handling, summary, and close (47). Over the past half-century, the Chinese government and medical societies have held a large number of such outreach programs to educate providers in remote regions. Such programs enhanced the knowledge and clinical skills of practitioners, especially in regions with very limited sources of information. However, educational outreach visits are very costly and could be biased if they are funded by bodies with conflicts of interest.

### 4.3. Local opinion leaders

Local opinion leaders are individuals or groups of people who are nominated by their colleagues as “educationally influential.” They spread their ideas efficiently through formal and informal channels within their community of impact. Proper assistance from local opinion leaders undoubtedly enhances the dissemination of evidence-based practices. According to a systematic review, involving local opinion leaders resulted in an ~12% improvement in professional practice compared to no intervention (48, 49). Both questionnaires and interviews have proven effective in identifying opinion leaders. Other strategies include self-designating methods, informant methods, and sociometric methods (50).

Opinion leaders could contribute to any classic or innovative approach to spread evidence. Their greatest value to professional education is their skill and level of experience, which facilitates their implementation of evidence in daily practice (51). Some opinion leaders might be potentially biased and underqualified, especially when there are conflicts of interest. In such cases, the education program must identify this situation and help qualified candidates improve their presentation skills.

### 4.4. Patient-mediated intervention

Patient-mediated interventions aimed to alter clinician performance through interactions with standard or real patients and the transmission of information from or to patients (52). Standard patients are those who are trained specifically to educate or assess the clinical skills of doctors and medical students. Standard patient intervention could improve clinicians' performance and patient outcomes (53, 54). A randomized trial showed that standard patient intervention could improve clinicians' smoking cessation counseling behaviors in practice (40% vs. 12%, *p* = 0.003) (55).

Beyond standard patients, there are many other forms of patient-mediated interventions including patient-reported health information, patient education, patient feedback, patient decision aids, patients or patient representatives, and patient-led training or education of healthcare professionals (52, 56). Patient-mediated interventions can achieve improvements in clinician practice, patient behaviors, and health outcomes (56). Patient-targeted interventions enhance patients' knowledge about their condition and support their role in decision-making, which in turn can encourage more active self-management. These interventions will prompt clinicians to provide healthcare following the guidelines. However, patient-mediated interventions encounter great resistance from the healthcare system. They require clinicians to give up their dominant roles in practice at a considerable cost of time (57).

Patient-mediated interventions are traditionally delivered face-to-face at or outside the practice site, either once or in a continuous system. In the post-pandemic era, these efforts employ a greater number of virtual meetings (58). Patient-mediated interventions are new and unfamiliar to China and most other developing countries and they may be costly to adapt.

### 4.5. Audit and feedback

The audit and feedback strategy use any summary of the clinical performance of healthcare over a specified period, aimed at providing information to health professionals to allow them to assess and adjust their performance (59, 60). According to a systematic review, audit and feedback could increase clinicians' compliance with desired practices by ~7% compared with no intervention (61). It works more efficiently among those with lower baseline performance and when feedback is delivered more intensively (60, 62). An audit and feedback strategy works best regarding targeted simple behavior changes rather than complicated ones (60). This is largely because fostering change with a high degree of complexity in the targeted behavior not only requires individual effort in daily work but also requires collective efforts at team and organizational levels (59).

### 4.6. Clinical decision-support system

In addition to sharing patient information, electronic health records also contain complete patient information that can help improve clinical decision-making (63, 64). A clinical decision support system (CDSS) embedded in an electronic health record is a new frontier of clinical practice guideline implementation (65). A CDSS automatically sends advice or reminders as well as background information to clinicians when triggered by a specific event (14). The CDSS sends reminders for those episodes at the appropriate time and improves clinical efficiency and quality of care. A systematic review found that CDSS increased the proportion of patients receiving desired care by 5.8% (65).

Although artificial intelligence has been involved in the development of the CDSS process, patients and clinicians are still the final decision-makers in most cases. All advice from a CDSS should, therefore, be evidence-based and clinically interpretable to support the judgment of the clinicians. The development of a CDSS is challenging because of its close interaction between medical and computer science. This requirement restricts the wide implementation of the CDSS, because it is difficult for the computers to understand the clinical practice guidelines. Ontology and its interpreting engines are thus recruited to develop computer interpreting guidelines and their affiliated CDSS (66–69). Guidelines with transparent supporting evidence facilitate this translation (25, 32, 70).

For example, a recently published guideline on sodium–glucose cotransporter-2 (SGLT2) and glucagon-like peptide-1 (GLP-1) receptor agonists for adults with type 2 diabetes contained all supporting evidence in its study pack (25). With interactive tools (MAGICapp or MATCH-IT tools), both clinicians and patients could quickly access the information and make shared decisions (32, 71). Through the process, clinicians can improve their clinical performance in a very efficient way.

## 5. Blended learning approach

Blended learning is a modern model of learning that integrates in-person or live face-to-face learning and online passive capture of knowledge with active and creative knowledge sharing in a constructivist model of learning (16, 18). Online learning can be synchronous (e.g., live e-learning class) or asynchronous (e.g., web learning modules) with later constructivist learning (18). In a typical blended learning project, the learners start with an online course that provides background information, basic knowledge, and upcoming interactive case materials, followed by an expert-supervised in-person workshop or training task that enhances the practical skills of the learners. Nevertheless, both the online course initiation and the later in-person workshop are flexible based on pragmatic needs. For example, constructivist elements may join the initial part of blended learning, especially in a clinical setting. After the pandemic of COVID-19, the in-person workshop is largely replaced by virtual meetings and discussions, especially in remote regions with Internet access.

Blended learning programs may be more effective than standard face-to-face lectures. One systematic review found that blended learning improved 40% of the knowledge acquisition of clinicians (72) and 30% of the self-reported clinical behavior (73).

One key advantage of blended learning is that it allows clinicians to learn on their own time and offers the convenience of not having to relocate (18). Most clinicians prefer blended learning for its convenience and minimized disruption to patient care, which is particularly important for doctors who work in rural areas and remote places (74). Online learning can also optimize the benefits of subsequent face-to-face sessions (74). However, some conditions should be taken into consideration during the development and implementation of blended learning (16, 74, 75). The cost of supporting equipment and training is the most critical challenge for institutes without particular experience (16). The proper quality control for the virtual lecture is also a guarantee of the full project (74).

## 6. Conclusion

Professional medical education is a crucial component in the evidence ecosystem (10, 76, 77). Trustworthy evidence merits dissemination with approaches that vary in benefits and negative impacts. Clinicians, the knowledge recipients, are taking more of a dominant position than ever before. Healthcare implementation scientists are moving their focus from information transactions to the active improvement of practical skills, resulting in the wide adoption of constructivist and blended learning activities. Nevertheless, traditional techniques continue to be used for their advantages of accessibility. Further implementation studies comparing different approaches may further facilitate the choice of these approaches in mobilizing evidence for clinicians in professional medical education.

## Author contributions

SL contributed to the design, concept, and finishing of this article. XL contributed to the concept and finishing of this article. All authors approved the submitted version.
